# Bioprinting in Vascularization Strategies

**DOI:** 10.29252/.23.1.9

**Published:** 2019-01

**Authors:** Mahboubeh Jafarkhani, Zeinab Salehi, Amir Aidun, Mohammad Ali Shokrgozar

**Affiliations:** 1 School of Chemical Engineering, College of Engineering, University of Tehran, Iran; 2 Tissues and Biomaterials Research Group (TBRG), Universal Scientific Education and Research Network (USERN), Tehran, Iran; 3 National Cell Bank of Iran, Pasteur Institute of Iran, Tehran, Iran

**Keywords:** Neovascularization, Three-dimensional printings, Tissue engineering, Tissue scaffolds

## Abstract

Three-dimensional (3D) printing technology has revolutionized tissue engineering field because of its excellent potential of accurately positioning cell-laden constructs. One of the main challenges in the formation of functional engineered tissues is the lack of an efficient and extensive network of microvessels to support cell viability. By printing vascular cells and appropriate biomaterials, the 3D printing could closely mimic *in vivo* conditions to generate blood vessels. In vascular tissue engineering, many various approaches of 3D printing have been developed, including selective laser sintering and extrusion methods, etc. The 3D printing is going to be the integral part of tissue engineering approaches; in comparison with other scaffolding techniques, 3D printing has two major merits: automation and high cell density. Undoubtedly, the application of 3D printing in vascular tissue engineering will be extended if its resolution, printing speed, and available materials can be improved.

## INTRODUCTION

One of the most important challenges in tissue engineering to form three dimensional (3D) functional tissues is vascularization. For *in vitro* survival, cells need a stable and flexible blood microvessel network to provide oxygen and nutrients[1-4]. To develop an extensive network of vasculature in engineered constructs, a multidisciplinary approach of vascularization, biomaterials engineering, and micro-fabrication techniques is necessary to imitate the cell microenvironment in *in vivo* conditions to induce the formation of mature blood microvessels and protect cell viability over time[5-9].

So far, great progress has been made in understanding the processes involved in angiogenesis. However, there is a long way to develop microvessels *in vitro*. To engineer a functional tissue *in vitro*, we need to realize the intricate biology of *in vivo* systems in order to mimic the major structural features found in the native tissue. For vascular tissue engineering, the main features are multi-scale, branched structure of vasculature, as well as the related diffusive and convective transport mechanism[10-12].

Vascular structure and morphology have a critical role in blood transfusion to various tissues, where the vessels’ architecture is highly dependent on specific requirements of the target tissue. With progress in more complex tissue engineering and the construction of larger 3D scaffolds, the preparation of an appropriate vascular network is highly demanded. The artery, vein, and lymphatic networks are the integral parts of a complicated tissue engineering process[13,14]. Most living cells in the body lie at a distance within the range of 100-200 μm from a single capillary to receive essential components such as oxygen and nutrients and dispose their waste products. This phenomenon is very essential for the cellular life[14]..

Vascularization and blood flow are two major challenges in organ engineering[15]. To eliminate the transport constraints, researchers have used proangiogenic factors such as vascular endothelial growth factor (VEGF) and basic fibroblast growth factor (BFGF) to induce blood microvessels generation. It has also been shown that the addition of endothelial cells (ECs) to the culture medium results in the formation of microvessels and eventually the formation of angiogenic sprouts in the engineered construct[16]. It should be noted that ECs and angiogenic factors do not produce perfusion constructs very quickly[17]. Bioreactors continuously transmit media from porous constructs and eliminate the need for arterial scaffolds and larger tissues[18]. However, these scaffolds and constructs are often without microvessels and stored outside the bioreactors for the survival of cells. Therefore, microfluidic-based vascular network preparation would be an option, but its application in larger physiological scales is challenging[17].

Another approach for vascularization is decellularization and recellularization of native tissues. This method not only provides the majority of the basement membrane and extracellular matrix (ECM) proteins but also generally maintains the original tissue structure[19]. It should also be noted that in reseeding process, the placement of cells is unpredictable and uncontrollable. Therefore, the cell positioning can be a problem in tissues containing several different types of cells. In this regard, 3D printing has recently been developed to control cell placement and the structure of vascular networks, as well as to form microchannels with suitable permeability[20-22]. The 3D printing has capability of constructing the layers of 3D structures, which offers a unique opportunity in tissue engineering to precisely control materials, cells, and every x, y, and z coordinate within the build volume. Using the 3D printing method in constructing scaffolds and complicated vascular networks, the perfusable channels can be constructed.

This review aims to present the current printing methods to induce microvessel formation. Although several *in vivo* approaches have been developed to stimulate vascularization, the present review emphasizes the methods that create extensive and perfusable vascular networks *in vitro* in 3D cell cultures.

### Blood vessel formation *in vivo*

**Fig. 1:**
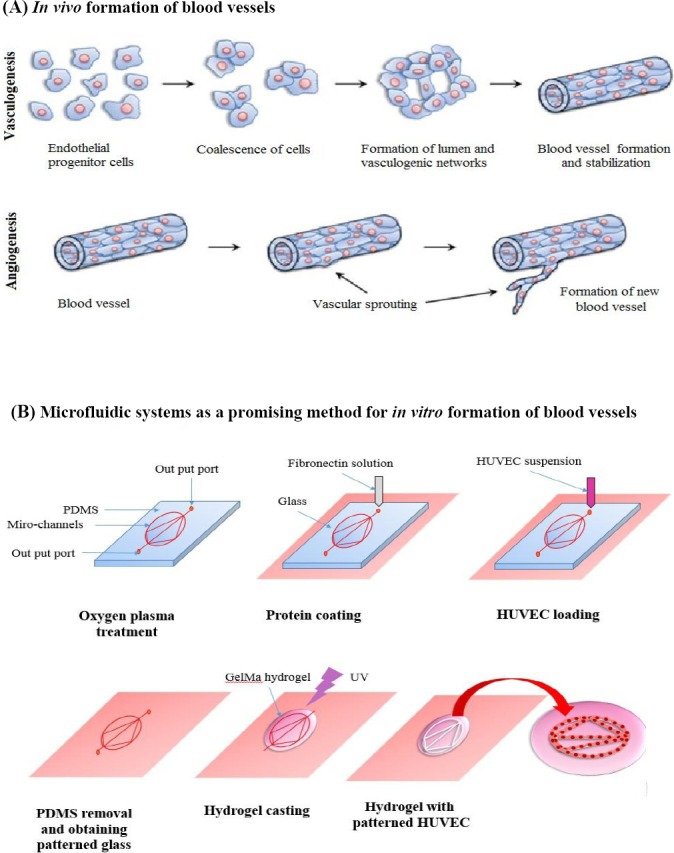
Blood vessel formation *in vitro* and *in vivo*. (A) A schematic process of *in vivo* blood vessels development, vasculogenesis and angiogenesis, the two main mechanisms of blood vessel development. Vasculogenesis gives rise to the early vascular network during embryogenesis. Angiogenesis remodels and develops the vascular network through biochemical cues containing budding tumors, hypoxia conditions, and growth factor gradient[24]. (B) A schematic process of fabrication to make microfluidic patterning and laterally confined microfluidic patterning devices using soft lithography method. In the first step, a microfluidic device (a patterned polydimethylsiloxane [PDMS] mold) and the glass were exposed to plasma treatment and bonded to each other. Then a solution of fibronectin was introduced to the device. In the next step, a solution of endothelial cells was entered to the inlet section of the system. When most of the cells attached to the surface (about 1 hour), the PDMS mold was separated to obtain a PDMS surface containing patterned cells. In the following, the PDMS mold was immersed in the culture media and then a layer of hydrogel was coated on the patterned surface[32].

For development of *in vitro* vascularized tissue successfully, deep understanding of the biological, physiological and functional aspects of blood microvessels formation is required to imitate the *in vivo* conditions[23]. In physiological condition, two main mechanisms of vascular formation have been described: vasculogenesis and angiogenesis (Fig. 1A). Vasculogenesis is a procedure in which endothelial progenitor cells generate new blood microvessels. This process has a fundamental role in embryo formation for the development of the first vascular plexus and heart[24]. In vasculogenesis, angioblasts are generated from the mesodermal stem cell differentiation into endothelial progenitor cells due to the presence of some biomolecules such as VEGF. Then angioblasts migrate to certain locations and form separate blood islands. In the following, blood islands join together and create a vascular plexus and ECs. Finally, the ECs migrate by different mechanisms (chemotaxis, haptotaxis, and mechanotaxis) and arrange to tubal structures and produce capillaries. The microcapillaries become more matured and bigger when the layers of smooth muscle cells and fibroblast are organized around microcapillaries[25].

Angiogenesis, as another mechanism of blood vessel formation, is a process in which the vascular networks are generated through the sprouting of ECs from existing vessels. In this mechanism, either ECs degrade the ECM and sprout from vessels and form capillaries or existing blood vessels split and create smaller capillaries[26].

### Blood vessel formation *in vitro*

Our understanding of the process of angiogenesis is largely comes from the available studies employing numerous *in vivo* and *in vitro* models[27]. However, due to technical issues related to *in vivo* assays, *in vitro* models can be considered as attractive alternatives. For instance, the *in vivo* visualization of neovasculature growth in animal models is a difficult task, and the management of chemical, geometrical and biological parameters during angiogenesis is challenging. These limitations confound the quantification features in these *in vivo* models. Thus, *in vitro* studies, including 2D scratch assays, barrier methods (Teflon stamp), chemical droplets, and planar microfluidics provide a useful, albeit simple, alternative to study the migration and morphogenetic assays of ECs[27]. More complex culture assays such as Transwell migration using modified Boyden chambers and the aortic ring assay are now routine[28]. However, technical limitations persist, Transwell devices lack a realistic microenvironment, and explant models suffer from issues of reproducibility and long-term viability. Alternatively, simplistic 3D engineered models have revealed important functions of single vessels under normal and pathological conditions, suggesting the need to move toward 3D *in vitro* assays[29].

Many models are now being designed to mimic *in vivo* geometries, empowered significantly by microfluidics-based techniques[30,31]. In addition to the ease of reproduction, cost-effectiveness and the use of flexible polymers such as polydimethylsiloxane (PDMS), microfluidics offers the potential to produce well-defined microscale geometries. They can also be designed to distinguish between the subtleties of chemokinetic and chemotactic effects[30,31]. For example, Rezaei Nejad *et al*.[32] combined microfluidic patterning techniques with surface microstructuring methods to generate planar multiscale protein, hydrogel, and cellular patterns. Simultaneously, they created microscale topographical features to restrict the patterned human umbilical vein ECs (HUVECs), as well as to control cellular growth and support capillarity based on creating continuous patterns (Fig. 1B). Their results indicated that this approach provides an opportunity to create certain patterns of cells and direct cells to grow vertically and generate 3D architectures of well-designed hydrogels. Combined with biologically-derived hydrogels, an increasing number of microfluidic designs have emerged to study 3D lumen and branched networks of capillaries through one of two approaches of predefined patterning or self-assembly, both of which will be discussed in the following sections[33].

### Simulating *in vivo* environments

For simulation of *in vivo* environments in healthy or pathological conditions, it is necessary to characterize the form and function of microvessels. There are a couple of parameters, including the number, average diameter, and length of engineered microvessels used to analyze the quality of microvessels network[34,35]. Meanwhile, many factors affect the formation of microvessels[35]. For instance, cell density changes the diameter and length of branches. Angiogenic factors (VEGF and S1P) stimulate the vascular network formation when co-cultured with other kinds of cells such as fibroblasts[35].

Immunofluorescence in vascularization studies is commonly applied to show EC phenotypes and their network connectivity. The analysis of CD31 expression levels and vascular endothelial cadherin content are the most common immunofluorescence techniques[36]. Zonula occludens-1 and EC polarity represent the indices of the tight junction between endothelial and vessel maturity, respectively[37,38]. Another factor needed for the simulation of *in vivo* environments is the evaluation of EC function, which can be performed by an ordinary thrombotic response upon contact to inflammatory mediators. As an early example, von Willebrand factor immobilization and prostacyclin release have been observed from the vessels inside the collagen gel[29]. ATP has also been shown to be able to promote a temporary rise in Ca^2+^, which increases nitrous oxide (NO) production in ECs. Both Ca^2+^ and NO directly influence vascular permeability[39,40]. Adhesion molecules such as intercellular adhesion molecule 1, melanoma cell adhesion molecule, and leukocytes adhesion as well as platelet accumulation, have been enhanced due to inflammatory cytokines[29]. It has also been reported that protein kinase C stimulation causes cytoplasmic Weibel-Palade bodies to increase the delivery of von Willebrand to ECs surface, where they bind to platelets permanently[29].

Numerous methods have been employed to evaluate the function of *in vitro* vasculature networks[41]. One of *in vivo* models commonly used as vascularization assay is based on Matrigel injection inside the abdominal wall of the body and development of host vessel in Matrigel. This assay has the diverse benefits of presenting a reproducible and direct method to measure angiogenesis. Matrigel, usually comprised of collagen IV, laminin, heparan sulfate proteoglycans, and entactin, polymerizes rapidly at body temperature after injection. Therefore, a solid biomaterial is formed in the body that is able to maintain its structure during the experiment time. During implantation, ECs invade through the Matrigel due to the delivery of angiogenic factor from the Matrigel[42]. However, it is indispensable to indicate the barricade to diffusion of different molecules and measurements of permeability.

### Role of 3D printing in vascularization

Nowadays, the simulation of vascular structures is a difficult task since fabrication techniques, especially for biomaterials, are still not well developed. The 3D printing is a new and an accurate method that usually uses materials such as resins and plastics to create 3D constructions layer by layer. This technique provides a great opportunity for scientists to fully control materials, cells, and their positions inside 3D structures and, therefore, develop the tissue engineering field. Until now, various methods, such as extrusion methods, selective laser sintering, and photolithography, have been applied for 3D printing[43,44]. These compatible methods are able to provide correct positions for cells in a bioprinting process. Although offering some benefits over cell-free approaches for biomedical engineering, bioprinting creates many challenges and difficulties for researchers to consider some important parameters, including materials biocompatibility, printing time, printing conditions, the choice of cell types, and different molecular factors[45-47]. In addition, some parameters such as flow rates, nutrient and oxygen diffusion, and cell behavior should be monitored precisely after a successful printing. The 3D printing also needs to be improved from different aspects, including resolution and cell handling ability. By addressing these complexities, the researchers would be able to fabricate the 3D functional engineered tissues, especially thick and complex constructs such as vascular channels, with appropriate biological and mechanical features for clinical applications[48].

### Extrusion printing

Extrusion printing is one of the most compatible, commonplace, and cost-effective bioprinting methods that uses bioink solution, including the monomers, cross-linker, and initiator molecules. This technique can be applied to a wide range of biomaterials with various properties and can print at very high cell densities[49]. Recently, a couple of investigators have utilized the extrusion method to print fugitive ink solution to generate vascular channels in a macroscopic scale by coating the channel surface with an endothelium layer via settling ECs through perfusion[2, 20, 50-54]. They applied cell-containing hydrogels and indicated enhanced cell viability while vascular networks were integrated. Their results showed that the regions close to the channels had significant differences in cellular viability compared to farther regions. Although well-matched with more polymers, the extrusion methods can limit the ultimate design and geometry of the polymer.

**Fig. 2:**
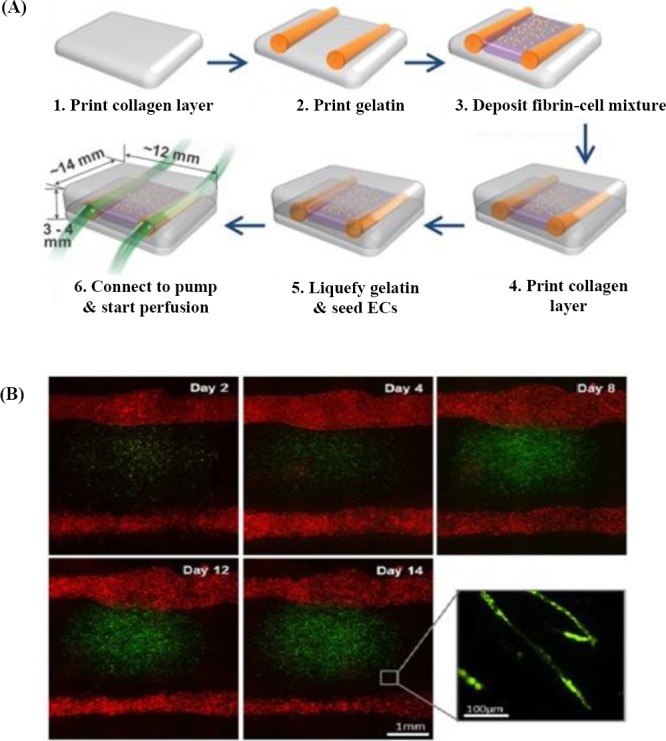
Extrusion printing method. (A) A schematic picture of two large channel structures and deposition of fibrin and cell, which were developed using the 3D bioprinter. (B) Florescent microscopic images of two large channels, and the mixture of fibrin cell placed between channels. Green dots show green fluorescent protein-ECs that were cultured within fibrin, and red dots indicate red fluorescent protein-ECs that were seeded on the two large channels[55].

Recently, Lee *et al*.[55] have developed a 3D printing model to produce capillary structures (lumen diameter of 0.5-1 mm) inside the fibrin network by a natural maturation procedure. They successfully achieved angiogenesis by sprouting ECs within a fibrin network loaded with other supporting cells such as normal human lung fibroblasts, hence presenting a feasible solution to attach capillary structures to the large perfused channels. In their model, there were two large channels, and capillary structures developed by sprouting EC were formed and linked to two vessels (Fig. 2A). They reported that their method provides a great opportunity in thick and vascular engineered tissues. The formation of vascular lumen coated by ECs enhances the diffusion rate of proteins and biological molecules (Fig. 2B). It is well known that native tissues contain a complex and well-defined mixture of different components. Mimicking this 3D intricate structure requires printing multiple cell types and ECM. To address this challenge, Colosi *et al*.[56] tried the capability of more than one bioink using microfluidic systems either individually or simultaneously. First, they optimized the composition of a biological bioink composed of gelatin methacryloyl (Gel-MA) and alginate. Subsequently, they developed a microfluidic printhead for bioprinting this low-viscosity solution. Cell-containing Gel-MA encapsulated in microfibers was chemically crosslinked using UV radiation, while alginate fiber acted as a structural template to maintain the printed multilayers. Ehsan *et al*.[57] presented a pre-vascularized tumor (PVT) spheroids model to study the early step of metastatic process, which includes blood vessel formation. PVT spheroids are composed of ECs, where the tumor cells are implanted in a fibrin hydrogel containing fibroblasts. Although their primary aim was to imitate the physiological mechanism of tumor, their model was a great instance of a 3D engineered vascular tissue model. They reported that the PVT model is able to support mechanisms of vessel formation via two mechanisms: improving strong sprouting angiogenesis inside fibrin and contiguous vascularization within the spheroids. In this model, cell mass printing in the pre-aggregation state was very negligible; therefore, there was a need for supporting printed mold with minor activity to aggregate cells. The reason is that a high-quality mold leads cells to form suspension instead of aggregation[58]. Li *et al*.[59] have used 3D printing based on extrusion method to fabricate vascular-engineered tissue. They used an extrusion system with dual nozzle to print two different hydrogels, one containing gelatin, sodium alginate, and chitosan with isolated rat hepatocytes and another consisting of a blend of gelatin, alginate, and fibrinogen comprised of rat adipose-derived stem cells [ADSC]) within a cooled space and stabilized in a solution of thrombin, CaCl_2_, and Na_5_P_3_O_10_. By using a dual nozzle printer, they could print a porous construction containing ADSCs embedded in vascular channels, which were surrounded by hepatocytes with a great resolution of 400 mm. The results indicated that ADSC differentiated into ECs-like cells, and hepatocytes secreted more albumin and fewer urea and alanine transaminases after two weeks. Their results also approved the great potential of this double nozzle printer to fabricate complex structures, which can be widely used in tissue engineering[59]. Another example of applying extrusion bioprinting is related to the study performed by Pati *et al*.[60] in which decellularized native tissues, including adipose, cartilage, and heart, were used to produce a bioink. They used decellularized tissue due to its excellent properties such as closely similarity to native extracellular matrix and suitable biological activity. By gelation of these biological solutions at 37 °C, after assembling,

The open porous constructs were obtained. Their results showed that the printing process of the solutions of decellularized tissue was performed successfully, and the level of cell differentiation was improved. Although having gained acceptance in printing different biomaterials, extrusion bioprinting still has some limitations such as low resolution and speed that needs to be improved[61]. Bertassoni *et al*.[50] developed a 3D vascular network of bioprinted agarose as a channel mold in different hydrogels such as Gel-MA, poly(ethylene glycol-co-lactide) acrylate (SPELA), poly(ethylene glycol) diacrylate (PEGDA), and poly(ethylene glycol) dimethacrylate (PEGDMA). They could successfully embed functional and perfusable microchannels inside the hydrogels and assess the effect of vascular network on the nutrient transport and cell viability. Using this method, they reported that the monolayers of ECs were formed inside the channels, and Gel-MA hydrogels could effectively improve the maturation of completely perfusable channeled structures with different shapes and geometries. More recently, Kolesky *et al*.[62] have developed a 3D printing approach to fabricate thick human tissues (>1 cm) with complete bio-mimicking *in vivo* constructs of ECM and embedded engineered microtubule structures and different cell types. They suggested that 3D vascularized models were actively perfused with long release of angiogenic growth factors (more than six weeks) to stimulate the differentiation of human mesenchymal stem cells to osteogenic lineage.

### Laser-based methods

#### Selective laser sintering (SLS)

**Fig. 3:**
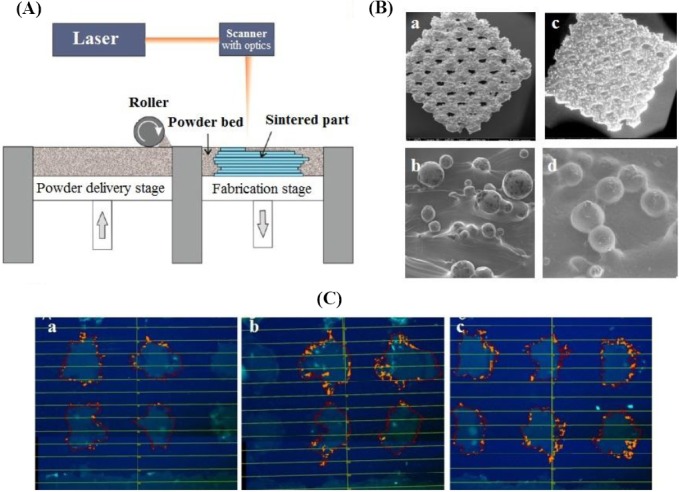
Selective laser sintering (SLS) methods. (A) A schematic picture of a SLS printer; (B) environmental scanning electron microscopy photos of three different samples, non-coated (a and b) and polycaprolactone-coated (c and d); (C) Wimasis image analysis ofv titanium implant’s crosssection with green fluorescent protein-osteoblasts seeded in different times: (a) one day, (b) three days, and (c) seven days.

SLS is a method based on powder materials instead of liquid materials and uses a high power laser for generation of 3D constructions (Fig. 3A)[63]. In this method, at first, a layer of powder is printed uniformly onto a surface, and then the temperature increased up to melting point of the powder. In the next step, a laser beam is used to enhance temperature in certain locations and incorporate the particles of powder material together. The other layers of the powder are printed in the same process. This feature of SLS contributes to print an extensive range of biomaterials from metals to polymers and ceramics[64,65]. However, contraction or deformity is the fundamental disadvantage of this technique. The resolution in this technique depends on the particle size of the powder, as well as laser power and its focusing precision. The work is underway to improve the features of SLS to print particles size less than 50 μm [66]. Matena *et al*.[67] have used a printing method based on laser power to produce a certain geometry with the pore size of about 250 µm for bone tissue engineering applications (Fig. 3B and 3C). Because bone tissue engineering needs fast vascularization, they used biomolecules such as VEGF and chemokine (C-X-C motif) ligand12 (CXCL12). The results of Live cell imaging showed that osteoblasts proliferated during seven days of culturing.

#### Stereolithography

**Fig. 4:**
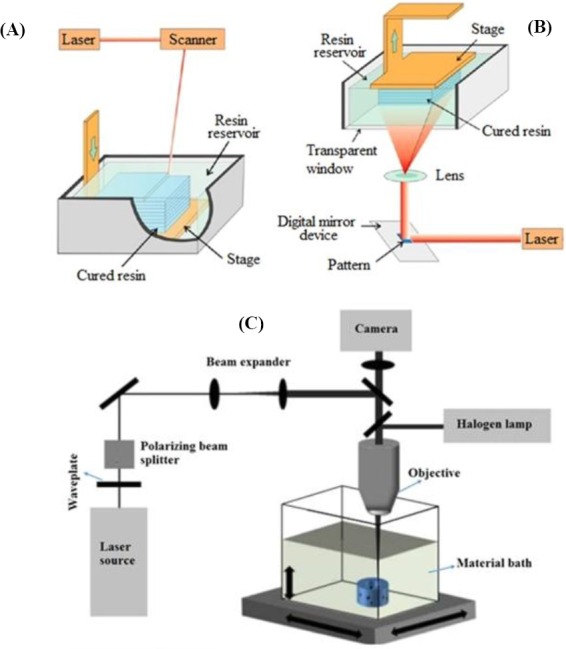
Stereolithography method of printing. (A) direct and (B) indirect or mask-based stereolithography. (C) An illustration of stereolithography to fabricate vessels in Huber et al.’s[71] study. A laser beam was polarized, expanded and then directed through a microscope objective.

Stereolithography uses laser power to crosslink or polymerize the bioink solution. Therefore, this method is limited to printing only photo-crosslinkable materials. In stereolithography, the curing time and deposited layer thickness are contingent upon the kinetics of the crosslinking process. Therefore, these parameters can be regulated by changing some factors, including the laser power, speed of scanning procedure, and initiator-to-monomer ratio photo. Laser stereolithography can be performed by two common methods: direct and indirect laser writing (mask-based lithography; digital light projection), as shown in Fig. 4A and 4B, respectively[68,69]. The direct method is comprised of a resin tank, UV light, computer, and mobile base. In addition to all the equipments mentioned for direct method, the indirect method contains a “mask” that has mirror form. There is another method of stereolithography that uses photo-polymerizable biomaterials (Fig. 4C). This technique is utilized generally for fabrication of larger constructions with a high resolution of 5 µm[70]. The Huber *et al*.[71] has also used stereolithography and printed a new photo-curable polyacrylate material to fabricate 3D synthetic vessels with the diameters of 2 mm and 300 µm wall thickness and with a range of different Young’s moduli (Fig. 4C). They have also used a rotating system for cell seeding to form a homogenous monolayer of ECs at the internal wall of the vessels. The results demonstrated that ECs maintained cell viability and adhered and arranged in the direction of medium flow.

#### Subtractive methods

**Fig. 5:**
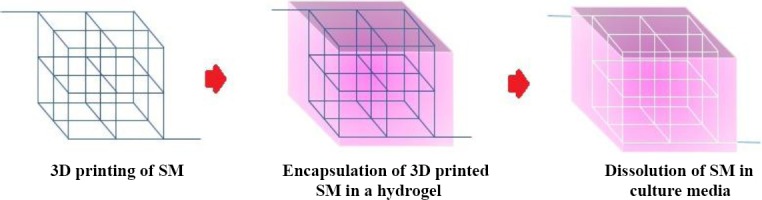
The process of the microchannel formation inside the gel using the 3D printing of sacrificial materials (SM). At first, a computer-based plan of a SM is fabricated using the 3D printing. Then this 3D construct was immersed in a solution of an appropriate biomaterial and certain cells. After gelation of the biomaterial, the 3D constructs were immersed in culture media, which flows inside the channel, dissolves SM, and creates microchannel within the scaffolds.

In addition to the methods mentioned above, subtractive methods have also been used to fabricate a network of vessels inside synthetic tissue constructs (Fig. 5). In this approach, a soluble material is printed. Using a solvent or increasing the temperature up to the melting point of the printed material, the channel is created. Miller et al.[54] have printed transparent and stiff solution of sugar glass containing carbohydrates and dextran, which were soluble in water. The main advantage of using this solution was its stability at room temperature, suggesting its appropriateness for many biomaterials comprised of different cells and compatible with an extensive range of biomaterials, including Matrigel, fibrin, collagen, alginate, etc. They have also observed that ECs rapidly form a layer on the channel walls. Their results of printing fibrin gels with hepatocytes revealed that the casting process occurred, and perfusable channels were created successfully; therefore, the production of albumin and cell viability was improved. Subtractive methods have considerable merits such as low handling of cells and using a widespread range of biomaterials[54]. More recently, Massa *et al*.[72] have developed a biomimetic 3D constructs including perfusable vessel via the sacrificial bioprinting technique for drug-toxicity study inside the engineered endothelial layer. They fabricated hollow microchannels of sacrificial material covered with a layer of HUVEC uniformly inside the construct composed of Gel-MA and HepG2/C3A cells. They reported that the presence of the HUVEC layer inside the biomimetic scaffold not only provided biomolecules permeability through 3D tissue construct but also improved the viability of HepG2/C3A cells.

Various investigations have used a combination of both extrusion methods with four printheads and subtractive materials to print few different biomaterials[73]. Kolesky *et al*.[73] have used Gel-MA-containing fibroblasts as the bulk construct and Pluronic F127 as the sacrificial material, which is soluble in water at 4 °C and can be removed by the mild flow of water. They could print fibers of Gel-MA inks and Pluronic F127 with a diameter of 45 to 500 µm in a PDMS chamber. After the photo-crosslinking process, Pluronic was dissolved in water, and microchannels, labeled with florescent conjugated ECs, were created. Their findings demonstrated that cell viabilities within the perfusable channels improved by up to 60–70%, directly after seven days of cell culturing. They also reported that this method provides an accurate positioning of cells inside the Gel-MA bulk, though its resolution still needs further improvement[73]. In addition, Kang *et al*.[74] have presented an integrated tissue-organ printer to fabricate human tissue constructs with different shapes. They could print cell-laden biomaterials and biodegradable polymers with suitable mechanical stability in certain patterns, which previously designed by a compute and fixed on sacrificial biomaterials. Besides, they could obtain the accurate shape of the tissue construct by this method. They have also utilized the clinical imaging data as a computer model of the anatomical defect and then translated them into a program to regulate the printer nozzels’ motions and to locate the cells correctly. They incorporated microvessels within the constructs to improve nutrient diffusion process and confirmed the capabilities of this method to form engineered tissues. Using this technique, they predicted that the fabrication of more complex and thick tissues would be feasible.

Vascularization is a major challenge in tissue engineering for fabrication of 3D complex tissues with a suitable function. The 3D bioprinting is able to provide a great opportunity to generate functional engineered tissues as well as vascular *in vitro* models. There are different strategies for printing biomaterials in 3D structures, including extrusion, SLS, stereolithography, and subtractive methods. Each method has its own advantages and disadvantages. An ideal bioprinting method should maximize the bioprinter abilities such as resolution and printing speed. Further, it should provide the possibility of printing a wide range of biomaterials that support the vascularization process. Meanwhile, the applied bioinks should both protect the cells from being damaged during the printing process and support vascularization. Nevertheless, for fabrication of a complex network of vessels using 3D printing, some technical limitations such as low resolution and long printing time should be considered to improve its application for tissue replacement. Indeed, high resolution is an important factor for printing suitable cell-laden materials in the patterns of small tubal structures within the shortest time. Ultimately, it is predicted that the combination of different approaches with bioprinting technique can offer more benefits to develop vascularized 3D constructs.
